# The general data protection regulation, the clinical trial regulation and some complex interplay in paediatric clinical trials

**DOI:** 10.1007/s00431-021-03933-3

**Published:** 2021-01-18

**Authors:** H. W. Dalrymple

**Affiliations:** Centre For Pediatric Clinical Development, PRA Health Sciences, 500 South Oak Way, Green Park, Reading, RG2 6AD UK

**Keywords:** GDPR, CTR, Paediatric clinical trials

## Abstract

Although a number of authors have commented upon the impact of the GDPR on clinical trial conduct, few have examined the specific setting of paediatric trials. Whilst the general principles are the same as those for adults, some additional considerations arise. The ages of consent relating to data privacy and clinical trial participation are different in a number of countries, but the distinction is often not recognised in non-drug trials. Accidental pregnancies in clinical trials always raise complexities, but these are amplified when the trial subject is a minor, and the processes described in clinical trial protocols rarely take account of GDPR requirements. This paper describes approaches which can be taken to ensure the rights of children are respected.

*Conclusion*: The conduct of paediatric clinical trials within GDPR requirements is quite possible provided authors think carefully when drafting protocols.

**Table:** 

**What is Known:** *•* *GDPR is applicable to clinical trials, including paediatric trials.* *•* *A number of challenges at the interface between the GDPR and CTR have been described.*	
**What is New:** *•* *The application of the GDPR to certain specific situations in paediatric trials does not appear to have been explored.* *•* *Three such situations are described and solutions offered.*

**What is Known:**

*•* *GDPR is applicable to clinical trials, including paediatric trials.*

*•* *A number of challenges at the interface between the GDPR and CTR have been described.*

**What is New:**

*•* *The application of the GDPR to certain specific situations in paediatric trials does not appear to have been explored.*

*•* *Three such situations are described and solutions offered.*

## Introduction

The General Data Protection Regulation (EU) 2016/679 (GDPR) [1] is the most important change in data privacy law in Europe for 20 years, and took effect in May 2018. The objective of the GDPR is to standardise and strengthen the protection of personal data across the EU and for other country’s data being processed within the EU, regardless of the processing companies’ locations outside of the EU. Accordingly, this is a global matter. Although the GDPR was not designed specifically for clinical research, its requirements apply in addition to those governing the conduct of clinical trials in Europe. Therefore, the GDPR governs the trial activities of all EU sites, as well as local and foreign sponsors and CROs processing personal data from EU subjects.

Both before and since the GDPR was implemented, a number of articles have appeared describing the impact of the GDPR on clinical trials [2], many anticipating the implementation of the Clinical Trial Regulation (CTR) (EU) No 536/2014 [3]. The purpose of a clinical trial of an investigational new product (IMP) is to gather reliable and robust data on the IMP [4], whilst the overall objective of the GDPR is to protect fundamental rights and freedoms of people and in particular their right to the protection of their personal data [5]. The CTR contains several Articles relating to the processing of personal data, e.g. Articles 37 (4) and (8), 41–43, 56 and 58. However, consent in the context of the CTR is a safeguard and not a legal basis for data processing, so a distinction must be drawn between the requirement for consent for a subject to participate in a clinical trial and the requirements for lawful processing of personal data under the GDPR.

The most thorough discussion of the interplay between the GDPR and the CTR has come, appropriately, from the European Commission [6]. However, none of these articles has specifically addressed the application of the GDPR to paediatric non-drug, non-device (e.g. methodology, biomarker and/or real-world) studies, nor to the occurrence of incidental pregnancies in paediatric subjects or their partners.

Commercially sponsored non-drug, non-device (ND-ND) disease studies are becoming increasingly common, for two reasons. One of these is the requirement within the 21^st^ Century Cures Act that all instruments used in pivotal clinical trials must be validated [7]. This is encouraging manufacturers to authenticate bioassays, biomarkers, patient-reported outcomes and other tools prior to deploying them in definitive efficacy studies with an IMP. Whilst the 21^st^ Century Cures Act is a US instrument and therefore not binding within the EU, it is nevertheless highly persuasive, as most manufacturers will wish their new products to be accepted by the FDA. The other is that new drugs and gene therapies are increasingly being developed to treat rare conditions for which basic information regarding incidence, prevalence, progression and standard of care is often lacking, and so prior to clinical trials, an information-gathering process is necessary to inform the development of the protocol. This process is subject to the GDPR, and when information is being collected from children, additional considerations come into play regarding the collection and processing of the information they provide.

Clearly, ND-ND studies are often conducted in adults, and subject and partner pregnancies occur in adult studies, so such settings raise issues regarding the authority to process data under the GDPR, but the clear capacity of adult participants to give legal consent for both trial participation and data processing distinguishes these trials from those in children. The aim of this article is to outline approaches which will help ensure that paediatric studies in which these circumstances arise are conducted in a GDPR-compliant manner.

### Methods

This review is the result of experience with over 40 paediatric clinical trials in which the author has been involved since the implementation of the GDPR, combined with an examination of the published literature and case law relating to the application of the GDPR to clinical research. No interactions took place with clinical trial subjects and no clinical or personal data were accessed as part of this review. Accordingly, ethics approval for this review was not required.

## Non-drug, non-device studies

Commercially sponsored studies of this type are becoming increasingly common, particularly in the treatment of rare diseases, and, by definition, most paediatric conditions are rare. Such studies take various forms. They include retrospective chart reviews, disease-monitoring programmes, natural history studies and registries. Some will entail the collection of additional information from patients, beyond that normally captured in medical notes, and some may request patients to complete questionnaires to explore the impacts of the condition and/or its treatment. Increasingly, such trials are becoming “interventional”, with blood or tissue samples being taken to identify biomarkers which may become indicators of disease severity or treatment effect. Such conditions may have neither an approved treatment nor an agreed standard of care, and the progression of the condition may not be documented in sufficient detail to enable the manufacturers of potential new treatments to provide compelling evidence of efficacy to regulatory authorities. Accordingly, ND-ND studies are increasingly being undertaken prior to IMP studies in rare conditions to generate such evidence.

According to the European Data Protection Board (EDPB), the processing of personal data is lawful if it falls under one of three legal bases, depending on the circumstances attached to a specific clinical trial: [8]

- A task carried out in the public interest under Article 6(1)(e) in conjunction with Article 9(2)(i) or (j) of the GDPR; or
- The legitimate interests of the controller under Article 6(1)(f) in conjunction with Article 9(2)(j) of the GDPR; or
- Under specific circumstances, when all conditions are met, data subject’s explicit consent under Article 6(1)(a) and 9(2)(a) of the GDPR.

For normal medical practice purposes, after consent for procedures relating to diagnosis or treatment, the legal bases for recording the patient’s data in the medical records are Articles 6.1(c) and 9.2(h) of the GDPR. However, these provisions do not cover the clinical trial setting, in which information in excess of that required for normal clinical care is routinely collected, and the information is shared with third parties, e.g. trial sponsors, contract research organisations and regulatory authorities, which are not directly involved in patient care. Although the further use of these data for research purposes may fall under Article 9.2(j) of the GDPR, in the author’s experience, the vast majority of commercially sponsored ND-ND studies conducted as part of an IMP development plan rely upon consent as the basis for processing personal data.

For studies involving minors, permission for personal data processing would normally be given by the holder of parental responsibility [9]. This is analogous to the situation which obtains in clinical trials of IMPs, where parental permission is required to enrol minors. Under Art. 8 (1) of the GDPR, the definition of a minor is an individual aged between 13 and 16 years, and within that range, member states are free to specify the relevant age in each country. As a result, the ages at which individuals are considered to have capacity to consent to the collection and processing of their personal data, and the age at which individuals are considered to have capacity to consent to enrolment in a clinical trial of an IMP, may be different (see Table 1).

**Table 1:** Ages of consent to data collection and processing and to clinical trial participation in Europe

Country	Consent age for clinical trial^a^	Consent age under GDPR^b,c^	Country	Consent age for clinical trial^a^	Consent age under GDPR^b,c^
Austria	14	14	Italy	18	14
Belgium	18	13	Latvia	18	13
Bulgaria	18	14	Lithuania	18	14
Croatia	NS	16	Luxembourg	NS	16
Cyprus	NS	14	Malta	18	13
Czech Rep	18	15	Netherlands	16	16
Denmark	18	13	Norway*	18	13
Estonia	18	13	Poland	18	16
Finland	15	13	Portugal	18	13
France	18	15	Romania	18	16
Germany	18	16	Slovakia	NS	16
Greece	18	16	Slovenia	18	16
Hungary	18	16	Spain	18	14
Iceland	18	13	Sweden	18	13
Ireland	16	16	UK	16	13

*NS* Not specified in published information

*Norway is not subject to GDPR and has introduced national legislation

^a^Lepola, P., Needham, A., Mendum, J., Sallabank, P., Neubauer, D., Saskia de Wildt, S. (2016). Informed consent for pediatric clinical trials in Europe. Arch Dis Child 2016;101:1017–1025. Available at https://www.ema.europa.eu/en/documents/other/informed-consent-paediatric-clinical-trials-europe-2015_en.pdf

^b^https://www.linklaters.com/en/insights/data-protected/data-protected

^c^https://www.betterinternetforkids.eu/en_US/web/portal/practice/awareness/detail?articleId=3017751

For trials involving IMPs, the requirements when a subject enrolled as a minor attains the age for giving valid consent are unequivocal: under Art. 32(3) of the CTR, consent must be obtained before the subject can continue to participate in the clinical trial, otherwise the subject must be withdrawn [10]. In contrast, the GDPR contains no such requirement, and ND-ND trials are not, of course, subject to the CTR.

Accordingly, three possible approaches exist for continued data collection in such a circumstance. One is to rely on the original (and hopefully adequately drafted) consent indefinitely and without regard to the data subject’s age, subject only to their withdrawal of that consent to the extent permitted by the GDPR. This may be legally defensible, but the viability of such an approach is untested, and the longer the period of enduring reliance upon parental consent, the greater the strain upon the ethics of doing so, even if the legal aspect is surmountable. For example, a 16-year-old minor enrolling in a ND-ND disease registry which is envisaged to collect data from each subject of a period of 10 years may be included based on the permission of holder of personal responsibility if the consent age under GDPR in that country is 18 years. It would seem unreasonable to continue to rely upon that parental permission a decade later when the subject is 26 years old, may be married and have a family, and have emigrated to another country.

Another approach is to seek to refresh the consent in a manner similar to that for trial participation, with the now-adult trial participant giving a renewed consent or validating the continued validity of the existing consent. Legally, this may be the safest, but it would also be the most burdensome route. The third option is to seek to apply a different legal basis than consent to the collection of data post-majority than when the subject was a minor. In many ways this may not be ideal, but it is perhaps justifiable on a case-by-case basis.

Given the clarity in the CTR regarding progression from minor assent to adult consent, the silence of the GDPR on this topic seems puzzling and provides an interesting contrast with another form of ND-ND observational research—biobanking—which does not come under a single, unifying regulation in Europe. In Italy, for example, calls are being made for guarantors or curators of paediatric biobanks to develop procedures for contacting donors when they come of age, so they can *inter alia* confirm or rescind the consent, have the samples destroyed and/or have the information eliminated [11]. A similar appeal has been made in the Netherlands [12]. The (non-binding) OECD-Guidelines on Human Biobanks and Genetic Research Databases include a requirement (Annotation 32) to obtain the child’s consent for ongoing storage and use of the material when the child reaches the age of majority [13]. The 2016 Recommendation of the Committee of Ministers to Member States on Research on Biological Materials of Human Origin stipulates that re-consent has to be obtained when a person attains capacity to consent [14]. The reasons behind treating the need (or not) for consent upon attainment of capacity for clinical trials, biobanking and data processing differently are not immediately apparent.

A somewhat related distinction between the CTR and the GDPR relates to assent. Whereas the CTR stipulates that assent should be sought from a minor who is capable of forming an opinion and assessing the information provided to him or her [15], the GDPR does not contain an explicit requirement for obtaining the assent of a minor before collecting and processing the minor’s personal data. However, the GDPR does require that any information and communication relating to data collection and processing, where it is addressed to a child, should be in such clear and plain language that the child can easily understand it [16]. The most prudent course of action, therefore, may be to seek the minor’s assent, particularly if the personal data collection period will continue beyond the point at which the child is considered to have developed capacity to consent or to withhold that consent. That way, the child is “bought in” to the data collection and processing activities, and may therefore be less likely to refuse consent when he/she attains the relevant age.

Obviously, clinical trials of new drugs entail the collection and processing of personal data as well as the conduct of the trial, and the discrepancy between the ages at which a minor attains capacity to consent to each of these is addressed in Recital 161 of the GDPR. This states that for the purpose of consenting to participation in scientific research activities in clinical trials, the relevant provisions of Regulation (EU) No 536/2014 (the CTR) should apply, i.e. the age of consent under the CTR takes precedence. Thus, in Belgium, for example, whilst a 13-year-old may consent to the collection and processing of his/her personal data as part of a ND-ND study, for an IMP study, parental permission is required for both trial participation and collection and processing of personal data relating to the trial until the individual is 18 years of age.

## Incidental pregnancy in paediatric trials of IMPs

The protocols for most clinical trials involving IMPs stipulate that upon discovery of an incidental pregnancy, i.e. a pregnancy which arises during the course of the trial and was unknown at enrolment, the study drug should be discontinued and the subject should be permanently discontinued from the trial. Few would argue the wisdom of such a requirement. Obviously, exposure in utero (EIU) to an IMP is a potentially worrying situation, and, understandably, most sponsors stipulate in protocols that the outcome of the pregnancy should be reported. Some go further and request a short developmental follow-up, commonly for periods of less than 2 years. Again, this is understandable and, many would say, entirely reasonable. However, the question then arises of the legal bases for such requests.

The consent or assent given to participate in a clinical trial lasts until the individual’s participation in the trial is complete. Completion could be at the final scheduled visit, at a post-treatment follow-up visit specified in the protocol (30 days is common), at an earlier time if the participant withdraws consent and leaves the trial or when the participant is discontinued from the trial for any reason. The permission given to collect personal data on the participant, by accessing the participant’s medical notes or otherwise, is not limitless, but, as discussed above, unless the investigator clarifies with the trial subject that in being discontinued from the trial she is also withdrawing consent to the processing of her data, the permission previously given by those with parental responsibility seems to endure.

This situation raises a number of practical problems. One of these is that the pregnant participant may no longer have any reason to continue to visit the investigator, unless the trial was for a gynaecological IMP. For example, if the subject had been participating in a metabolic disease trial, the investigator would most likely be a metabolic disease physician. If the subject has been withdrawn from the trial, per protocol, and has returned to the care of her primary care practitioner, the specialist has no reason to see her again. The “controller”, for GDPR purposes, is now the primary care physician rather than the specialist, so once the pregnancy is complete, how would the investigator legitimately become aware of its outcome?

The participant herself may volunteer that information, and arguably, the holder of parental responsibility who gave permission for the participant to enter the trial may be empowered to volunteer that information insofar as it relates to the (former) trial participant. However, if the participant has been discontinued from the trial, she is no longer subject to CTR age criteria, and GDPR age norms take effect so, depending on the age limit in the relevant country, the holder of parental responsibility may no longer have the right to share their daughter’s personal information with a third party, specifically an investigator who has no enduring obligation to or relationship with the former trial participant.

An additional complexity may also arise. The pregnant participant can, of course, tell whomever she desires about her pregnancy, including the investigator. However, if the participant has been discontinued from the trial, and is under the age of consent as defined under GDPR, it is questionable whether the sponsor can use information provided by the participant in the absence of parental permission.

Article 28(2) of the CTR explicitly refers to the situation where consent may be sought from a trial subject for the use of personal data outside the trial protocol for future scientific purposes. This would require a valid legal ground under GDPR Art. 6, which may or may not differ from the legal basis of the primary use, and would take account of GDPR Art. 5(1)(b) which provides for a presumption of compatibility of purposes, i.e. the assessment of the safety of an IMP (particularly in a vulnerable patient, such as a minor) [17], subject to the conditions in GDPR Article 89(1), whereby further processing is carried out for purposes of scientific research. Where consent (GDPR Art. 6(1)(a)) is used as a legal basis for the processing of personal data for a secondary use, that consent may be sought at the beginning of the clinical trial (the first use); this form of consent must be distinguished from the informed consent in the context of the CTR. If the aim of using the data for further research outside the protocol arises after the clinical trial has been completed, the sponsor must seek specific consent [18].

Clinical practice has been defined as activities designed solely to enhance the well-being of an individual patient, which may entail the deployment of innovative or non-validated therapies. In contrast, clinical research comprises activities designed to develop or contribute to generalisable knowledge, consisting of theories, principles or relationships which can be corroborated by accepted scientific observation and inference [19]. By participating in a clinical trial as an investigator and enrolling a patient as a participant, the physician has moved from clinical practice—recommending a course of action, adjudged as being in the best interests of the patient—into clinical research, in which the obligation has become one of ensuring that no harm comes to the patient. Many trial participants conflate these two settings. Whilst a general obligation of confidentiality exists amongst medical professionals to enable free sharing of patient data for clinical practice purposes, that may not cover the situation of a primary care practitioner making known the outcome of a pregnancy to another medical practitioner whose only interaction with the individual was whilst she was a participant in a clinical trial some months earlier.

The developmental follow-up of the new-born raises additional challenges. Whilst the permission given by the girl’s parents to allow her to enrol in the clinical trial could, arguably, become a basis upon which the outcome of an incidental pregnancy could be made known to the investigator, the girl’s parents are unlikely to be the “holder(s) of parental responsibility” for the new-born, as defined in the GDPR, unless the new-born has been adopted or warded by the participant’s parents. If the former trial participant has attained the age at which GDPR confers the right to consent to the collection and processing of personal data, and is “the holder of parental responsibility” for the new-born, then she can authorise the collection and processing of data relating to the new-born.

One potential route to authorisation to collect and process the data from the new-born is the law courts. It seems plausible that the courts may consider an application under GDPR Art. 9.2(h), that processing is necessary for reasons of substantial public interest, i.e. the detection of possible teratogenicity, or GDPR Art. 9.2(i), that processing is necessary for reasons of public interest in the area of public health, such as… safety of health care and of medicinal products. However, these also appear to be untested. Clearly, if EIU has resulted in a latent disability, the sooner that is recognised, the better for the child, and for society. The collection and processing of the information would impose no risk or burden upon the new-born, and so the needs of society could be met without disadvantaging the new-born. Attractive though this may be, the question remains regarding who can petition the court for such a ruling. The sponsor seems an unlikely candidate: due to GDPR considerations, the trial participant is effectively anonymous to the sponsor. The investigator’s responsibilities relate to the clinical trial, although it may be possible to construct a “best interests” argument to support an application by the investigator, and the investigator would legitimately know the identity of the former participant. Even though a minor, the trial participant herself could in theory raise such an application.

In some countries, mechanisms pre-dating the GDRP exist which may be relevant here. For example, in Belgium, Germany or the Netherlands, a guardian can be appointed who would provide permission instead of the minor parent [20]. Furthermore, in Belgium or the Netherlands, a minor parent can, in some circumstances, apply to be emancipated from her own parents/guardians so that as an “adult” she could provide permission to collect and process developmental data relating to the new-born.

The lack of clarity regarding the authority for the collection of personal data for children of minor parents has been addressed by few academic authors. One legal commentator described the situation in the UK as “doctrinal incoherence” [21]. A paper, published before the GDPR was implemented, from a multi-national group of authors drawing on experience from the USA, Europe, Latin America and Africa made a number of recommendations to facilitate appropriate access and equity related to the participation of children of minor parents in clinical research, but did not address the legal issues relating to this [22].

The simplest solution may lie in the drafting of the original protocols which seek to enrol minors into clinical trials. Were these protocols to contain a statement to the effect that, should a trial participant be found to have become pregnant, she will be asked then to participate in a non-interventional (non-drug) study, that would provide the framework for follow-up until the outcome of the pregnancy is known. Such an approach would be consistent with CTR Art. 28(2) and the relevant consent could be sought at the beginning of the clinical trial which would constitute the primary use of the data collected. Many parents are likely to be resistant to this approach, and it may prove necessary to approach the parent(s) for specific permission for their daughter to participate in a separate non-interventional, non-drug study once the pregnancy is known.

Assent and consent to participate in such a study could be sought in the same way and under the same conditions as any other non-drug study, as described above, and the study would have the advantage of Ethics Committee approval. If, at the time of delivery, the trial participant had the capacity to give permission for collection of developmental data from the new-born, then that could be sought at the appropriate time, again subject to Ethics Committee approval. If the projected delivery date occurs before the trial participant attained that capacity, then that would allow a period, normally of some months, to collect data up to and including delivery, whilst in parallel exploring options to continue to collect and process data relating to the new-born post-natally.

## Pregnant partners of paediatric participants in IMP trials

Despite warnings in information sheets and assent documents, female partners of male trial participants do become pregnant and, in some cases, trial sponsors understandably wish to follow the pregnancy of a trial participant’s partner. This raises numerous privacy issues. The partner has, almost certainly, neither seen the information sheet nor signed an assent or consent form; she may, in fact, be completely unaware of the clinical trial in which her partner is a participant, and if the partner is a minor, her parents also may have no knowledge of the trial. Thus, no one involved in the trial has any consent to contact, or collect personal data relating to, the partner.

Whilst a trial participant is quite at liberty to disclose that he is about to become a father, the first time that the Investigator should become aware of any information relating to the pregnant partner is when the trial participant produces evidence of her consent to disclose that information. Whilst the participant may disclose such information verbally, should the investigator record, store or process that information, it becomes subject to the GDPR. Accordingly, before the investigator can do that, or contact the partner, or her parents, the investigator needs permission to do so or to make contact with the partner (if the partner is of an age to give consent under the GDPR) or, somewhat paradoxically, from the holder of parental responsibility, if the partner is under the GDPR age of consent. This paradox raises another data privacy issue: if the investigator requires permission from the partner’s parents to contact the partner, then the partner’s privacy has already been irretrievably compromised. In such circumstances, the investigator can provide an assent/consent form as appropriate to the participant and/or his parents, and ask the participant and/or his parents to request the necessary permission from the partner and/or the holder of parental responsibility for his partner, as appropriate. The investigator must be satisfied that the partner is above the GDPR age of consent before approaching her directly, rather than via the holder of parental responsibility.

Clearly, such situations will be sensitive and may raise legal issues beyond the GDPR. The trial participant cannot be compelled to disclose the name of or any other information relating to his partner. Similarly, the partner cannot be compelled to agree to disclose any personal information. The legality of collecting information indirectly, via the trial participant, is questionable. The same is true of any attempt to collect such information from the pregnant partner’s primary care physician. Although the father of a child may be as entitled as the mother to share information regarding the outcome of the pregnancy, including sex, weight, length, APGAR score and information relating to congenital abnormalities, the father cannot share information relating to the mother’s health or condition without her approval, or that of the holder of parental responsibility for his partner, if the partner remains a minor as defined under the GDPR. If the trial participant is not the holder of parental responsibility for the new-born, then the investigator cannot record, store or process that information under the GDPR. Collecting further data relating to the new-born as part of a developmental follow-up may be considered a non-drug, non-interventional study, and in many countries, the permission of both parents will be required, provided they have attained the GDPR consent age. If this is not the case, then following the arguments presented above, the collection of further information may be possible only via the law courts, perhaps again relying upon GDPR Art. 9.2(h) or 9.2(i) that data processing is necessary for reasons of substantial public interest, i.e. the detection of possible teratogenicity or to ensure safe healthcare. However, this also is untested, and the courts may be somewhat less inclined to support such an application when the EIU has been indirect.

## Conclusions

This article has sought to review three specific situations in which, based upon the author’s personal experience, awareness of the obligations under the GDPR is often less, perhaps understandably so, than that of the CTR requirements. In all three situations, specific consideration of the GDPR requirements during the protocol drafting process should allow adequate time to plan contingency strategies in the event of pregnancy and ensure the correct approach to consent is taken if the protocol is for a ND-ND study involving minors.

Pharmaceutical sponsors of clinical trials are well-versed in the relevant regulations and legislation appertaining to clinical trials of IMPs. It is therefore hardly surprising that sponsors will seek to work to similar standards in trials which do not involve IMPs, and will often assume that the age at which a trial subject has capacity to consent to processing of personal data will be the same as that for clinical trial participation. It seems important that the rights of those included into these studies be upheld, and recognising the relevance of the GDPR age limits in this respect is relevant. It also seems pertinent to note that legislative compliance in Europe remains a responsibility of the trial sponsor rather than the Ethics Committees, in contrast to that in the USA, where the IRBs fulfil this obligation together with trial sponsors.

For ND-ND studies, the simple recognition that the age limits applicable to IMP studies are not relevant to other types of trial is all that is required to ensure legislative compliance in this regard, provided protocols are worded appropriately. For some sponsors, the notion that an individual aged 13 years, in some countries, may give consent for personal data collection and processing without the permission of the person with parental responsibility, will be thought-provoking, and explaining the exigencies of the GDPR to such a young trial participant will be challenging. However, most sponsors are familiar with generating assent documents for clinical trial participation which are comprehensible to participants of that age, so the challenge should not be insuperable.

Incidental pregnancies in paediatric clinical trial participants are remarkably rare. The author of this article is aware of around 5 cases in his 30 years of clinical trial experience of around 1000 clinical trials, and of a slightly greater number of partner pregnancies. Once again, however, the rights of those participants must be respected, and the application of GDPR in this setting is more complex than in many others in the clinical research arena. Careful planning and forethought can reduce the challenges in these situations, by considering such situations in the trial protocol and prospectively defining the courses of action to be taken, recognising the GDPR requirements. In that way, the continued collection and processing of information which will contribute to the overall safety database for the IMP will be possible. The potential approaches described in each of these settings are captured in a summary form within Table 2.

**Table 2:** Possible approaches to ensuring GDPR compliance in trials involving minors

Situation	Possible approaches
Non-drug, non-device trial	1. Ensure that the protocol selection criteria stipulate that permission to participate will be requested from the holder of parental responsibility, unless the participant has attained the age of capacity to consent, according to national law. 2. Ensure that this topic is addressed during investigator training.
Incidental pregnancy in a minor trial subject	1. Describe within the protocol the processes which will be followed if: (a) The subject is under the GDPR age of consent at the time of delivery; (b)The subject is over the GDPR age of consent at the time of delivery; (c)The subject attains the GDPR age of consent during the follow-up period. Ensure these processes are described within the information given to parents and female participants prior to trial participation. 2. Create provision within the protocol for a sub-protocol to describe the post-natal follow-up of any incidental pregnancies, including: (a)Provisions for parental consent and subject assent, and for conversion of assent to consent once the subject attains the relevant age; (b)Definition of the process by which information will be collected, and by whom—this may require qualification of a different investigator from the one involved in the parent trial; (c)Ideally, employing the same sub-protocol in all protocols in a development programme will support the consistent collection and analysis of data. Ensure this provision is explained in the information given to parents and female participants prior to trial participation. 3. Consider whether a basis other than consent could be employed to authorise the collection of data in a post-natal setting. 4. Ensure that the processes to be followed are described during investigator training.
Pregnancy in a minor participant’s partner	1. Identify prior to trial commencement appropriate legal sources within each country which may advise regarding the appropriate process to follow in each country, specifically that for making contact with a minor partner. 2. Define within the protocol the process which will be followed should this circumstance arise, and ensure it is described within the information given to parents and male participants prior to trial participation. 3. Draft and have approved by Ethics Committees the documents which will be used to inform a pregnant partner or her parents: (a) Regarding the clinical trial; (b) Explaining the need for follow-up; (c) Describing the information required and how it will be collected. 3. Ensure that the processes to be followed are rapidly shared with investigators should the need arise.

Two other avenues may help to ensure compliance with GDPR requirements in these settings. The first is investigator training. Investigators are normally familiar with regulations relating to clinical trials, but may be less acquainted with the GDPR requirements for non-drug, non-device trials. Sponsors can support site staff by providing relevant training prior to study commencement, and in the construction of information sheets and assent/consent documents. The nuances around data collection during pregnancies in minor trial participants or their partners in a clinical trial setting, as opposed to a conventional medical one, may also be unfamiliar to investigators and their staff, and creation of appropriate training by sponsors which could be delivered if and when appropriate should be helpful.

The second potential avenue is via the reporting of trials. Clinical study reports must contain a statement relating to compliance with Good Clinical Practice and/or the CTR, although that is not relevant to non-drug, non-device trials. Perhaps the introduction of a requirement for a similar statement for GDPR requirement, which would be applicable to all clinical trials, would help to foster compliance.

## Data Availability

N/A

## Abbreviations

CTR: Clinical Trial Regulation (EU) No 536/2014
EIU: Exposure in utero
GDPR: General Data Protection Regulation (EU) 2016/679
IMP: Investigational medicinal products
